# Making of a pediatric urologist

**DOI:** 10.4103/0970-1591.36711

**Published:** 2007

**Authors:** K. V. Satish Kumar, S. N. Oak

**Affiliations:** Department of Pediatric Surgery, TNMC and BYL Nair Hospital, Mumbai Central, Mumbai - 400 008, India

**Keywords:** Pediatric urology, pediatric urologist, pediatric surgeon

## Abstract

The million-dollar question that we wish to address in this article is who should be the specialist for urological problems in children in India. What are the special attributes of a pediatric urologist? The answer to this question is far from simple and straightforward. Dogmatic rules do not provide a functional solution. While one has to admit that in India pediatric urological problems will be dealt with by urologists, as well as by pediatric and general surgeons, one has to also accept that lacunae do exist in the current curriculum and the methodology of training.

A pediatric urologist by the virtue of definition would be a specially trained surgeon who would deal with urological diseases of children from newborn to adolescence (0–14 years). (In some instances e.g. exstrophy, renal transplant and continent procedures the age of treatment may even exceed up to 16 and above). He has the experience and the expertise to treat a child. Children are not miniaturized adults and newborns are not miniaturized children. Their physiology is different, they cannot always express their problems, answer questions and may not be cooperative during clinical examination.

## ATTRIBUTES OF A PEDIATRIC UROLOGIST

A pediatric urologist with his experience in dealing with children knows how to examine and treat them in a comfortable, conducive and cooperative manner. He often uses equipments specially designed for children. Most offices where children are treated are arranged and decorated keeping the child-patient as a focus. If you want to treat them well, you have to understand them well.

Pediatric urology thus demands much more than balloons and tubes, stents and slings. It is in fact a whole process of innovative philosophy and structuring strategies.

A general urologist may not be fully conversant and competent to manage complex urological problems in children. As of view, the urologist who undergoes postgraduate training leading to a Master's degree in urology or an equivalent Diplomate by the National Boards, has limited exposure to pediatric patients. In places where the medical Colleges have Departments of Pediatric Surgery, the case load gets divided and secondly, the large number of Pediatric Urology problems are so severe and life-threatening that unless they are properly managed during the pediatric age, the children do not reach adulthood to get the services of the general urologist. Even if they do, irreparable damage has already occurred.

Pediatric surgeons currently treat all surgical conditions in children except the complex neurosurgical and cardiac defects. Pediatric surgery is an age-based specialty. Surgical care of children is frequently claimed to be best in the hands of a trained pediatric surgeon who understands the physiology and the effects of surgical stress in the milieu interior of a child. However, the fact remains that the general surgeons are performing almost 70–80% of pediatric surgical procedures as the pediatric surgical specialists are not available in all centers or the medical colleges.[1] The curriculum for training pediatric surgeons involves a large amount of urological work. To qualify as a pediatric urologist one must devote minimum of 50% of his or her practice to urological problems in children. It is also an accepted fact that childhood surgical deaths are more when surgeons mix up pediatric and adult practice.[2] In a study done by Snow *et al.*, on ureteroneocystostomy done on 184 children by general urologists and pediatric urologists it was found that the hospital charges were significantly less ($1095 per patient) and the complication rates were fewer when pediatric urologists did the procedure. The same study showed that the time required to complete a bilateral procedure was much more for a general urologist as compared to a pediatric urologist. The complication rates were lesser when urologists with appropriate subspecialization performed the procedures. The probable reason for this is that operations performed frequently by an individual surgeon will be quickly and safely accomplished.[3,4]

Better pediatric patient surgical outcomes have been documented under the care of a pediatric subspecialty trained surgeon regardless of discipline.[5]

“The aim of pediatric surgery is to set a standard, not to create a monopoly” quoted Dennis Browne,[2] the Father of Pediatric Surgery. The purpose of pediatric urology is to safeguard the interest of the superspeciality for the benefit of development of advanced surgical care in children. It is well said that the level of development of civilization in the society is a direct reflection of the significance it has given to achieve the care of health of children in that country.[2]

In 1959 the Platt report made a number of recommendations about caring for children in hospital. The six main principles were: 1. Child and family-centered care, 2. Specially skilled staff, 3. Separate facilities, 4. Effective treatments, 5. Appropriate hospitalization and 6. strategic commissioning.[2] In order to accomplish these goals in urological problems in children, we should create specialist pediatric urological care centers.

Most pediatric urological procedures can be done in one sitting, but some diseases like exstrophy bladder, proximal hypospadias, undescended testis etc, may need staged procedures. Hypospadias surgeries can be done by urologists, pediatric surgeons and plastic surgeons, if proper expertise, training and facilities for child care are present. Though single-staged repair can be safely and effectively performed even in patients with the most severe penoscrotal hypospadias, such single-stage approaches are best left to the experts in the field. It is worse to create a hypospadias cripple than not operating him at all. Neonates differ from other age groups in many aspects including body surface area, thermoregulation organ immaturity, fluid and calorie requirements and should best be left to the neonatologist and neonatal surgeons. Neonatal urology should be managed by a pediatric urologist in a tertiary care center. A specialist center should see about 100 new neonatal surgical cases a year and a minimum of 60 cases to remain a viable center.[2]

Tertiary centers should have complete autonomy, so that these can deliver advanced patient care, including transplant programs, endoscopy training and quality teaching and need-based clinical and experimental research.

All neonatal urology surgeries, surgeries in child with complex anomalies and renal transplants need to be done in tertiary care centers.

## INDIAN SCENARIO

Population > 1,10,00,00,000

Metro-based (30%)

Village-based (70%)

No. of teaching centres of pediatric surgery-26

Total number of medical colleges -200

Enough for 10-15% pediatric population

Every year about 30 qualified pediatric surgeons

Total number of qualified and registered pediatric surgeons: 981 (Indian Association of Pediatric Surgeons)-981 (IAPS)

Total number of qualified and registered urologists: 1700 (Urological Society of India)

Overall ratio-1 pediatric surgeon for approximately 3, 50,000 children

## CAN WE EVOLVE A CURRICULUM?

Training of the pediatric urologists should be planned to ensure that their training needs are met. The rotations should be designed to give trainees exposure to urologists who practice different specialized interests at least two years work with the appropriate specialists. Training needs must have priority over service requirements if the next generation of pediatric urologists is to be provided with specialist skills. Attempts need to be made to define approximate numbers of trainees for the subspecialties and those making a career choice should be advised appropriately. The proportion of trainees in the shortage-subspecialties needs to be increased and a mechanism for this has to be established. When all factors are considered, it is obvious that high-quality care with minimal complications will be most cost-effective and inpatient care least expensive if it is delivered by subspecialists.

## WHAT WILL BE THE ROLE OF MAS IN PEDIATRIC URO-TRAINING?

The emergence of laparoscopy and retroperitoneoscopy on the horizon of pediatric urology has further complicated the issues of training. Not many senior surgeons who at present head the various departments of urology and pediatric surgery are adequately exposed to minimal access surgery in children. Therefore the rotation of the students will also have to include a term of at least three months with adult and pediatric laparoscopy setups.

## IN ORDER WE SHOULD WE DEVELOP CENTERS OF EXCELLENCE

We would propose that training in pediatric urology should be done in centers where pediatric surgeons, urologists and anesthesiologists exclusively involved in pediatric surgical procedures are working together. Such a center should have all disciplines for children under one roof.

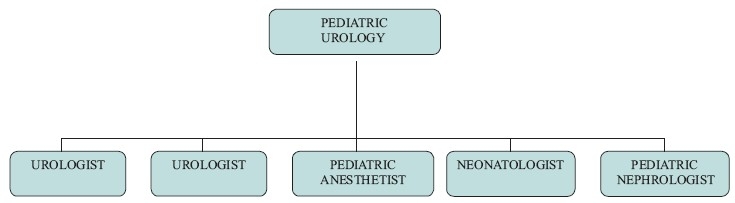



## PEDIATRIC RENAL TRANSPLANT?

The Pediatric Kidney Transplant Team includes specialists in:

**Table:** 

Pediatric nephrology	Medical ethics	Transplant coordination
Urologic surgery	Nutrition	Child life
Psychiatry and psychology	Social work	Education
Pediatric and home dialysis	Specialized nursing	Specialized pharmacy

A Pediatric Kidney transplant specialist can be one who has undergone fellowship training in transplant surgery in a transplant center with the following criteria.

The program must provide adequate volumes of various transplant procedures and have a formal structure of didactic and clinical training in place to be accredited. Kidney Fellowship done in a center which does 60 transplants per year should be a minimum requirement and the Fellow should have been actively involved in 30 transplants as primary surgeon.

## ANTENATAL SCREENING, COUNSELING AND INTERVENTIONS?

Antenatal diagnosis is made for only 10–15% anomalies. Screening for anomalies should be done by pediatric radiologist or one who dedicates most of his time to pediatric radiology.

Antenatal counseling should be done by a team of obstetricians, pediatric urologist and radiologist and social worker. Prenatal interventions are still in infancy. They can only be done in specialized centers or at tertiary care centers by experienced personnel who have been legally licensed to carry out prenatal interventions.

## SHOULD INDIA FOLLOW THE WEST OR IS IT MORE SENSIBLE TO EVOLVE A MODEL OF OUR OWN?

Pediatric Urology has established itself as a separate subspecialty and is an essential component of the Intercollegiate exams both in Pediatric Surgery (FRCS Peds) and Adult Urology (FRCS Urol) and the Board exams in the USA.

In the US, Pediatric urologists are medical doctors who after four years of medical school and one year of surgical internship have at least three additional years of residency training in general urology and at least two additional years of fellowship training in pediatric urology (2007 AUA Society for Pediatric Urology). The Senate of the Royal Colleges of Surgeons has recommended that general surgeons undertaking the care of pediatric surgical patients in district general hospitals should have undergone a six-month period of training in an approved pediatric surgical unit in Year 3 of training or above. In addition, these “general pediatric surgeons” should participate in audit and maintain continuing education in pediatric surgery.

In India the aim of the specialist center should be to treat patients efficiently with a consistently high level of care. There should be limited number of specialist centers of excellence, each surrounded by centers of competence serving local communities. They can then work together to provide the facilities and expertise that is demanded of a children's surgical service at both the local and regional level.

## CONCLUSION

It is evident from the existing scenario that there is scope for improvement and the common forum should contribute to the betterment of the faculty. We are therefore here not to compete but to coexist and cooperate.

‘It is difficult to say that is impossible, for the dream of yesterday is hope of today and reality of tomorrow’ Dr. Robert H.Goddard (Quote from world press.com)

## References

[1] Gupta DK (2005). President's speech. J Indian Assoc Pediatr Surg.

[2] Arul GS, Spicer RD (1998). Where should pediatric surgery be performed?. Arch Dis Child.

[3] Singh JC (2006). Are we ready for subspecialization and group practice in India?. Indian J Urol.

[4] Brent W, Snow MD, Cartwright PC, Young MD (1996). Does surgical subspecialization in pediatrics provide high-quality, cost-effective patient care?. Pediatrics.

[5] Snow BW (2005). Does surgical subspecialty care come with a higher price?. Curr Opin Pediatr.

